# Arsenic, Cadmium, and Lead Levels in School Meals and Their Risk Assessment in Municipalities in Bahia, Brazil

**DOI:** 10.3390/foods13101500

**Published:** 2024-05-12

**Authors:** Larissa da S. Santos, Fabiana F. Chagas, Martinho G. Dinis Martinho, Erival A. Gomes-Júnior, Mariângela V. Lopes Silva, José A. Menezes-Filho

**Affiliations:** 1 Graduate Program in Food Science, College of Pharmacy, Federal University of Bahia, Av. Barão de Jeremoabo, s/n, Ondina, Salvador 40170-115, Bahia, Brazil; nut.lari@gmail.com (L.d.S.S.); mmartinho@unilurio.ac.mz (M.G.D.M.); erival.amorim@ufba.br (E.A.G.-J.); 2 Graduate Program in Nutrition and Health, School of Nutrition, Federal University of Bahia, Rua Basílio da Gama, s/n, Canela, Salvador 40110-907, Bahia, Brazil; fabianacofranca@hotmail.com; 3 Laboratory of Analytical Chemistry, University of Bahia State, Rua Silveira Martins, 2555, Cabula, Salvador 41150-000, Bahia, Brazil; mlopes@uneb.br

**Keywords:** school meals, toxic metals, inorganic contaminants, schoolchildren, risk assessment, food security

## Abstract

Background: School meals represent a significant supply of nutrients for children in Brazil, especially those in conditions of social vulnerability. Objectives: This study aimed to assess the levels of arsenic (As), cadmium (Cd), and lead (Pb) in meals served in public elementary schools in four municipalities in the state of Bahia, Brazil, and assess the risk posed to children’s health. Methods: Ninety-six samples were collected from 16 schools, freeze-dried, and subjected to microwave-assisted digestion. The As, Cd, and Pb levels were determined by graphite furnace atomic absorption spectrometry. The risk assessment was based on calculating each element’s hazard quotient (HQ). Results: None of the samples reached or exceeded the tolerable levels for the elements analyzed. Pb was the metal that obtained the most significant result, reaching maximum levels of 39–157 µg·kg^−1^. Conclusions: No element exceeded the PTWI proposed by JECFA; thus, the toxic metal content in school meals poses a negligible risk to children’s health.

## 1. Introduction

Food was, and still is, a matter of individual and collective concern, considered a fundamental need to guarantee survival, and is the responsibility of individuals, communities, and states [1].

Various chemical substances are present in our foods, such as those essential for maintaining health, such as carbohydrates, lipids, protein, vitamins, minerals, and additives, as well as a diversity of contaminants from various sources. Mycotoxins, pesticide residues, and heavy metals are the most prevalent and of great concern due to their toxicity. The lack of any of these nutrients or the excessive presence of toxic substances in food can pose a risk to human health, especially for children, as they are still in the growth phase [2]. Therefore, the presence of physical and chemical contaminants in food and microbiological substances potentially harmful to human and feedstock health is today one of the major concerns of consumers, and the process of industrialization of animal and plant production represents a significant impetus for the increase in these contaminants in foods, such as school meals.

Because of this need, the School Meals Campaign was set up in Brazil in the 1950s, and after several improvement processes, it was renamed the National School Feeding Program (PNAE) in 1979. The PNAE aims to contribute to growth and biopsychosocial development, learning, school performance, and the formation of healthy eating habits. The program also aims to promote food and nutrition education and provide meals that cover part of the nutritional needs during the school term, serving mainly socially vulnerable children and offering a fundamental source of nutrients and energy [3,4].

The implementation of this program in the country took place at the same time as the term food safety (FS) emerged in the world. Although FS mainly guarantees access to food and nutritional quality, it also encompasses its biological and sanitary quality [5,6]. Although most incidents related to food contamination are associated with biological risks, food contamination by inorganic contaminants has become a growing problem, mainly due to the development of new technologies and the inadequate disposal of toxic waste by large industries [1,7].

Children may suffer repeated exposure to these toxic elements, as they often put their hands to their mouths, and even in small quantities, they can trigger health risks [8,9]. Children also have physiological factors that facilitate intoxication by these elements, as they are in the midst of a growth phase. In addition, they consume more significant quantities of food per kilogram of body weight when compared to adults, absorption in their gastrointestinal tract is faster, and their renal system is still immature [9]. Metals such as cadmium (Cd) and lead (Pb) have an affinity for the kidneys and bones [10], respectively, having long half-lives of 10–30 years, making them significant exposure sources for unborn children due to remobilization during pregnancy and breast-feeding [7].

The International Agency for Research on Cancer (IARC) has classified arsenic (As), organic and inorganic, as a Group 1 human carcinogen [11]; despite being a metalloid, it is classified as a toxic element regardless of its mass [12]. Also, according to the IARC, lead is a probable human carcinogen (Group 2), and an increase in blood concentration from 0.5 to 5.0 μg/dL can reduce children’s IQ scores by up to 8.6 points [13,14,15,16]. Concerning Cd, Chandravanshi et al. [15], in a review study on the toxic effects of this inorganic contaminant, recorded several findings for neuropsychological changes in children, especially in the early stages of life, which can cause cognitive dysfunctions, cancer, and nephrotoxicity.

Several studies carried out in Brazil and around the world have shown the presence of inorganic elements such as As, Cd, and Pb in foods consumed by the general population and in foods for children, mainly in rice, rice products, cereals, fish, crustaceans, and chocolate [17,18]. Silva et al. [19] analyzed samples of chocolate from different brands from Brazil and Germany, with the highest reported contamination being the contaminant Cu (138 µg·g^−1^ ± 3), followed by Pb (27.9 µg·g^−1^ ± 3.8). On the other hand, regarding concentrations of Cd, Pb, and other elements in samples of canned peas, corn, pineapple, and peaches from Brazil, in intact and damaged packaging, under normal storage conditions, Pb and Cd were not found in any of the samples, which were considered to be in compliance with food contaminant laws [20].

Few studies have quantified the presence of inorganic elements in meals served to school children. A study conducted in Chile found As levels below the Provisional Tolerable Intake (PTI) established by the FAO in meals from 65 schools [21]. In Brazil, research was carried out with meals offered to preschoolers, and the results indicated that there were no concentrations of As and Cd that could, at the time, cause harm to children [22]. Another study conducted in Brazil analyzed the presence of As, Cd, and Pb in samples of meals from daycare centers in São Paulo, where high levels of blood Pb had previously been found in children from these institutions [23]. Moreover, no values higher than those stipulated by the European Union were found in that study.

Therefore, research must be carried out to understand and generate data on the state of chemical contamination by inorganic elements in the meals served to schoolchildren. Given the above, this study aimed to assess the levels of inorganic elements (As, Cd, and Pb) in school meals offered in four municipalities in Bahia, Brazil, and the risk of exposure to these toxic metals.

## 2. Materials and Methods

A descriptive study was carried out with a quantitative approach, collecting data and samples of school meals from public primary education institutions in 16 schools in four municipalities in Bahia, Brazil.

### 2.1. Study Area

**Figure 1:**
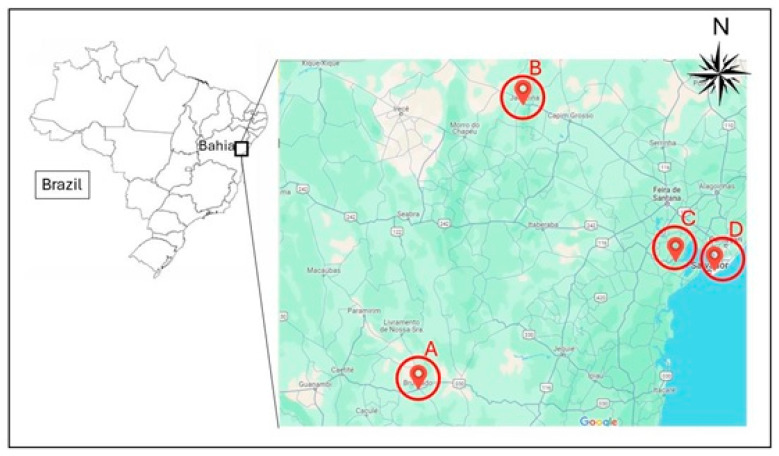
Geographical location of the study municipalities. A—Brumado; B—Jacobina; C—Jaguaripe; D—Salvador (Capital of the state of Bahia).

This study is a follow-up of the work carried out by França et al. [24], who investigated the composition of essential elements in meals served to schoolchildren in the municipalities of Jaguaripe, Brumado, and Jacobina (Figure 1), state of Bahia, Brazil. In these municipalities, the authors collected 72 samples of the meals served in four schools in each of the municipalities in the two school semesters of 2016. The mineralized samples were stored at room temperature in the Toxicology Laboratory of the Federal University of Bahia (UFBA), Brazil.

In this current work, we collected school meal samples from four elementary schools in Salvador, the state capital of Bahia, in 2019. Since we aimed to evaluate the possible impact of environmental contaminants on socially vulnerable communities, we obtained a list of elementary schools from the Salvador Municipal Department of Education. Thus, the schools included were located on the city’s outskirts, known as Subúrbio Ferroviário, where an impoverished population lives, including those four with the most enrolled students that year.

Data from the 2015 school census show that the municipality of Brumado had 84% of schoolchildren enrolled in municipal schools served by the PNAE, Jacobina had 71%, Jaguaripe had a total of 95% [24], and Salvador had 60.7% of children enrolled in municipal educational institutions [25]. Thus, there is a significant representation of children served by the PNAE who are potentially exposed to any contaminants in the meals served. On the other hand, all schools selected in this study participate in the PNAE program. Supplementary Table S1 shows more detailed characteristics of the municipalities included in this study.

### 2.2. Sample Planning and Collection Technique

The same technique used by França et al. [24] was used to assess students’ daily intake. Identical portions of food and drink served to the study population was collected on three alternate days in the first and second semesters of 2019 in four schools in the municipality of Salvador, in addition to the samples collected in the previous study. 

The visits took place without prior scheduling, allowing for reliable collection of the actual conditions of the schools. The samples were collected and packed in an unrefrigerated isothermal box and transported to the Toxicology Laboratory, where they were weighed on an analytical balance (CP2245 Sartorius^®^, Gottingen, Germany) and homogenized in an industrial blender (regardless of their consistency). They were then packed in polypropylene tubes, previously decontaminated with 10% HNO_3_, and stored in an ultra-freezer (Quimis^®^, Q315U-33, São Paulo, Brazil) at −33 °C until mineralization processing.

### 2.3. Microwave-Assisted Mineralization

After being removed from the ultra-freezer, a small amount of each sample was subjected to moisture analysis using the methodology of the Adolf Lutz Institute (IAL). Two grams of the sample were weighed into a previously dried and tared porcelain capsule. The capsule was placed in an oven at 105 °C until it reached constant mass [26]. It was cooled in a desiccator with silica gel until it reached room temperature. Afterward, the sample was weighed. Moisture percent content was calculated by [(m*i* − m*f*)/m*i*] × 100%, where m*i* is the mass of the sample (g) before drying and m*f* is the final mass of the sample (g). The rest was subjected to freeze-drying (Liotop L101 Freeze-dryer, São Paulo, Brazil); freeze-dried material was ground in a mortar using a porcelain pestle until a homogeneous powder was obtained. Next, about 0.2500 g of each sample was weighed on an analytical balance (CP2245 Sartorius^®^, Gottingen, Germany) directly into the Teflon^®^ tubes of the digester oven and recorded to the fourth decimal place. Subsequently, 4.0 mL of ultrapure concentrated nitric acid (J.T. Baker^®^, Philipsburg, PA, USA) and 1.0 mL of hydrogen peroxide (Suprapur Grade, Merck^®^, Darmstadt, Germany) were added. 

After 15 min at room temperature, the Teflon^®^ tubes were sealed and placed in the microwave-assisted digester oven (CEM, Mars 6^®^, Matthews, NC, USA), using the standard program for food digestion, whose specifications were as follows: power 900–1800 W, temperature 210 °C, pressure (psi) 800, ramp time 20–25 min, digestion time 15 min, and cooling time 15 min.

After cooling to room temperature, the mineralized samples, including reagent blanks and certified reference materials (CRMs) used for quality control purposes, were volumetrically transferred to polypropylene graduated tubes and volumetrically filled to 10 mL with ultrapure water (Merck-Millipore^®^, Billerica, MA, USA).

### 2.4. Determination of Toxic Elements

Graphite furnace atomic absorption spectrometry (GFAAS) was used to determine the inorganic contaminants using a Varian spectrometer (Spectra AA 240FGZ, Mulgrave Victoria, Australia). A calibration curve was used for Pb at concentrations of 0.2, 0.4, 10, 16, and 20 μg/L at a wavelength of 283.3 nm and a lamp current of 10 mA. For Cd, a curve was used at concentrations of 0.05, 1.0, 2.0, and 4.0 μg/L at a wavelength of 228.8 nm and a lamp current of 4 mA. For As determination, the standard addition technique was used, and the equipment was programmed to add to each sample and control two concentration values (30 µg/L and 60 µg/L) from a standard solution of 100 µg/L at a wavelength of 193.7 nm and a lamp current of 10 mA. Considering the moisture of the samples, the results were expressed in wet weight (*w*/*w*). All samples were analyzed in duplicate, expressing the average values in µg·kg^−1^.

### 2.5. Analytical Quality Assurance

For quality assurance purposes, two certified reference materials (CRMs) were used: apple leaves (NIST-1515) and spinach leaves (NIST-1570a) (National Institute of Standards and Technology, Gaithersburg, Maryland, USA). The method’s precision was calculated based on the coefficient of variation of eight replicates (four in one week and four the following week), analyzed on the same apparatus by the same analyst. Accuracy (Relative Error—RE) was calculated as the difference between the value obtained from the CRM and the certified value, and then divided by the certified value from the CRM. Finally, the result was expressed as a percentage after being multiplied by 100. The reagent blanks were analyzed in eight replicates to calculate the limits of quantification (LOQ) and detection (LOD), which were calculated considering the average concentration of the blanks plus ten times the standard deviation (SD) for the LOQ and the average concentration of the blanks plus three times the SD for the LOD, as performed by França et al. [24], who followed national and international quality assurance guidelines [27,28].

### 2.6. Risk Assessment of School Meal Consumption

The exposure assessment in this study is based on possible adverse effects on human health (non-cancerous) due to exposure to As, Cd, and Pb resulting from the consumption of the food served, based on the calculation of the risk quotient (*RQ*) [17,29,30] from Equation (1):

Equation (1). Risk quotient (non-cancer) associated with food consumption.

(1) $$RQ=\frac{Ef\times ED\times C\times FIR}{EfD\times Wab\times AT}\times 10^{-3}$$

where *Ef* is the frequency of exposure (days/year); *ED* is the duration of exposure (years); *FIR* is the average daily intake per person (g/day); *C* is the concentration of the toxic element in school meals (μg kg^−1^); *Wab* is body weight (kg); *AT* is the average exposure time to non-cancerous substances (365 × age); and *RfD* (or *EfD*) is the oral reference dose (mg·kg^−1^/day) (Cd = 1.0 × 10^−3^ μg·g^−1^/day, Pb = 4.0 × 10^−3^ μg·g^−1^/day and As = 0.3 × 10^−3^ μg·g^−1^/day) [31].

For this calculation, we considered the data obtained by this study and by the most recent Family Budget Survey (SOF) [32] in three different scenarios, described in Table 1 and in scenarios 1 (S1), 2 (S2), and 3 (S3), which correspond, respectively, to elementary school students (a period lasting nine years), secondary school students (a period lasting three years), and the overall average number of years that students served by the PNAE remain in school (12 years), under Law No. 9.394 of 1996 [33]. 

In these scenarios, the duration of exposure (*ED*) corresponded to the time that the students must remain in school, the average daily intake (*FIR*) corresponded to the median weight of the food obtained in the schools in the two school semesters, the frequency of exposure (*Ef*) considered the number of school days during 2019 (202 days), and the body weight (*Wab*) used was the median of that reported in the SOF (2008–2009) [34]. There is no reference dose (maximum acceptable limit) for oral Pb, so 4 × 10^−3^ µg·kg^−1^ was adopted, as followed in other studies [17,30].

### 2.7. Statistical Analysis

Microsoft Excel^®^ was used to build the database and calculate the moisture content. SPSS statistical software, version 20 (SPSS Inc., Chicago, IL, USA), was used for statistical analysis. The results of the analysis of the concentration of toxic elements in the food samples were described with median, minimum, and maximum values and interquartile ranges (IQRs) for each municipality for the two sampling periods. The non-parametric analysis of variance Shapiro–Wilk test was used to evaluate the differences in the median concentrations of inorganic elements between the municipalities, and the Wilcoxon test was used to identify possible statistical differences between the collection periods with a 95% confidence level. The analytical methodology was adapted following the recommendations established in the guidelines of national regulatory authorities DOC-CGCRE-008 (2016) of INMETRO (National Institute of Metrology, Quality, and Technology) and RDC no. 166 of the National Health Surveillance Agency [35]. Studies of linearity, sensitivity (LOD and LOQ), precision, and accuracy (as recovery) were performed.

## 3. Results and Discussion

### 3.1. Quality Assurance

The parameters used in this study’s methodology to guarantee analytical quality are shown in Table 2.

The methodology’s precision and accuracy align with current standards, as the European Commission recommended [36], with the values obtained being below 10%. Also, according to the legislation in force in the country, the parameters agree that the difference between the expected value for the MRCs and that obtained in the analyses was well below the acceptable limit of ≤20% [35]. The method’s sensitivity was also satisfactory, with the LOD and LOQ being sufficiently low and within the recommended standards for trace metal analysis. Therefore, the method’s validation is in accordance with the guidelines for all the elements analyzed.

### 3.2. Levels of Toxic Metals in School Meals

In this study, the average weight of the portions served to schoolchildren was 192.5 g/day, ranging from 150 g/day to 235 g/day in Brumado; 194.1 g/day, ranging from 133 g/day to 260 g/day in Jacobina; 236 g/day, ranging from 185 g/day to 255 g/day in Jaguaripe; and 257 g/day, ranging from 57.5 to 488 g/day in Salvador. These values were used to calculate the hazard quotients.

The medians, interquartile ranges (IQRs), and maximum and minimum values calculated for the three elements (As, Cd, and Pb) are described in Table 3, Table 4, and Table 5, respectively.

#### 3.2.1. Arsenic

The highest values found for As were observed in the schools of the municipality of Jacobina (5 µg·kg^−1^) in the second semester and in Salvador (4 µg·kg^−1^) in the first and second semesters (Table 3). The meals in this study were analyzed entirely. The differences in weight observed in Salvador may, in part, be related to the composition of the meals collected. As these are different municipalities, despite belonging to the same state, the school meal program is determined by municipal decision-makers. Moreover, there are eating habits that are typical of each location. Therefore, as the meals in this study were analyzed integrally, some specific ingredients may have influenced the observed differences. Nevertheless, in Brazil, the existing legislation only covers limits for food items and not for the total diet [37]. As in this study, the meal as a whole was analyzed; thus, it was impossible to verify each ingredient’s percentual contribution. A study by Maihara et al. [22] in schools in the municipality of São Paulo, Brazil, also found concentrations below those permitted by the legislation, in which daily As intake values were obtained with a median of 64.7 µg·kg^−1^.

According to the EFSA, it is estimated that dietary exposure to inorganic As in children is around 2 to 3 times higher than in adults due to various factors, including hand-to-mouth behavior and consuming more food per kg body weight per day (kg.bw/day) compared to the adult population [7].

**Figure 2:**
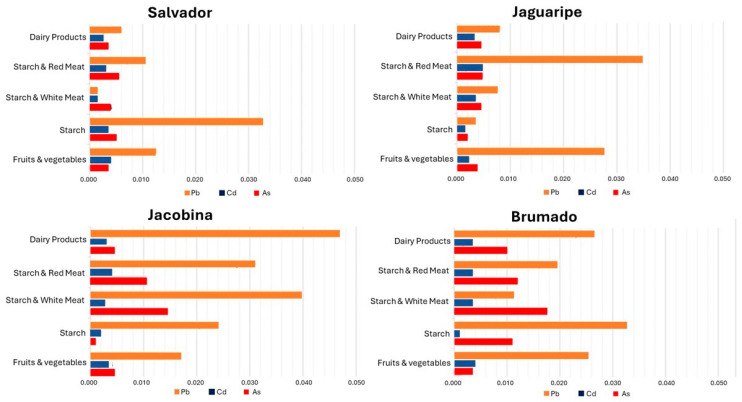
Metal content in µg/kg (w.w.) in school meals served in the four municipalities classified by the major food groups.

The risk of toxic effects from dietary consumption of arsenic increases, especially when the population consumes a greater quantity of cereal and starchy foods, particularly rice or seafood, which can be seen in the municipality of Jacobina, where a value of 1 µg·kg^−1^ was found in the first semester and 5 µg·kg^−1^ in the second semester. This difference can be explained by the fact that, in the second semester, the supply of starchy preparations was higher than in the first, especially those containing rice, such as rice pudding and rice with meat and vegetables [38,39]. Figure 2 shows metal contents by food groups (milk and milk products, starch and red meat, starch and white meat, starchy food only, and fruits and vegetables) in the four studied municipalities.

None of the preparations obtained in this study contained fish or seafood, which justifies the values of total As below those reported in other studies, such as the work carried out by Roma et al. [40] in Italy and Bastías et al. [21] in Chile, where the levels of total As were higher. Although the maximum limit established by the Brazilian national regulatory agency for most foods is between 100 and 300 µg·kg^−1^, none of the meal samples reached this limit [37].

JECFA does not have a safe PTWI value for arsenic exposure. At its last meeting, a PTWI of 15 µg·kg^−1^ was established. The maximum value of As found in this study was 10 µg·kg^−1^ in the second half of the year in Salvador and Jaguaripe and 11 µg·kg^−1^ in the second half of the year in Jacobina. If the stipulated weight value for population S1 was 25.4 kg, and that for S2 was 51.8 kg, the exposure values for Salvador and Jaguaripe would be, respectively, 0.010 and 0.09 µg·kg^−1^ for S1, 0.050 and 0.045 for S2, and for the second semester in Jacobina the exposure values would be 0.084 µg·kg^−1^ for S1 and 0.041 µg·kg^−1^ for S2; thus, in this hypothesis, the exposure values would not exceed the PTWI proposed by JECFA. It is worth noting that this figure would only reflect a single meal eaten by the population.

#### 3.2.2. Cadmium

The values found for Cd are shown in Table 4, with the values for the second semester in Jacobina, Jaguaripe, and Salvador being the highest, at 5 µg·kg^−1^, 4 µg·kg^−1^, and 4 µg·kg^−1^, respectively. Considering the national regulatory agency [37], all analyzed meal samples were below the maximum acceptable limits (300 µg·kg^−1^).

Like As, Cd has rice and wheat as the leading oral deliverers of contamination in the human diet. Rice, along with other cereals, legumes, and meat products, is the basis of Brazilians’ meals and one of the primary sources of exposure to Cd [7,13,41]. Many of the samples collected in the schools for this study contained rice in their composition, such as rice pudding and rice with meat or chicken, as depicted in Figure 2. 

Again, corroborating with the literature already cited, the difference in values between the first and second semesters in Jacobina is because, in the second semester, the samples collected had a more significant amount of starch and meat products in their composition. Despite the low values found for Cd, it is essential to note that this toxic element has a long half-life. Several authors have reported harmful effects on children’s health, primarily cognitive and behavioral effects, demonstrating an inversely proportional relationship between Cd levels and IQ, as well as significant associations between Cd levels and learning disorders in children and adolescents aged 6 to 15 [42,43,44].

#### 3.2.3. Lead

Brumado and Jacobina presented the highest levels of this inorganic contaminant, at 42 µg·kg^−1^ and 38 µg·kg^−1^, respectively.

As shown in Figure 2, the Pb contents were higher in the municipality of Jaguaripe than in other municipalities because the school meal samples had as ingredients foods of animal origin (poultry meat) and stew, which is generally accompanied by other types of meat, including pork. According to EC Regulation No. 1881/2006, of 19 December 2006 [45], these foodstuffs have the highest levels of Pb among the foodstuffs analyzed from meals served in schools, diverging from the fact that these studies identified higher concentrations of As and Cd [40,44,45]. For Roma et al. [40], exposure to lead measured in the diet was <7 µg·kg^−1^, while Nacano et al. [46], evaluating meals served to schoolchildren in São Paulo, obtained values between 1 and 33 µg·kg^−1^. Although the results show that Pb medians are below the maximum limit proposed by the national guidelines for most foods, some meal samples presented Pb concentrations above those limits, which should be considered a potential risk for children.

The analysis of this toxic element in foods, especially those offered to children, is essential, as even small amounts can trigger various health effects ranging from speech, hearing, hyperactivity, IQ reduction, and growth and development disorders, among others [47,48].

JECFA stipulated a PTWI for Pb of 25 µg·kg^−1^ of body weight per week; the maximum values of Pb found in this study were 137 µg·kg^−1^ in the second semester for Salvador and 157 and 137 µg·kg^−1^ for the first and second semesters for Jaguaripe, respectively. Following the hypothesis based on the scenarios established, Salvador’s exposure values for S1 and S2 would be 1.38 and 0.68 µg·kg^−1^, respectively. For Jaguaripe, in the first semester, the exposure values for S1 and S2 would be 1.45 and 0.71 µg·kg^−1^, respectively, and in the second semester, the exposure values would be 1.27 and 0.62 µg·kg^−1^ for S1 and S2, values higher than those found by Leroux et al. in a study carried out in kindergartens in São Paulo, where the total value of Pb for children was 0.18 µg·kg^−1^ [23].

All the municipalities showed higher concentrations of Pb in the second semester. One possibility highlighted could be a variation in the food served. In Brumado, where the value for the second semester increased sixfold, the meals served contained more fruit and vegetables with starch in their composition, similar to the observations of previous studies in Brazil. Vasconcelos-Neto et al. [49] reported values ranging from 20 to 30 µg·kg^−1^, with the highest values found in meals containing fruit and vegetables.

No statistically significant differences were found between most of the medians of the samples taken in the two semesters (*p* > 0.05), except for Cd, which differed in the municipalities of Salvador (*p* = 0.011), Brumado (*p* = 0.025), Jaguaripe (*p* = 0.011), and Jacobina (*p* = 0.03), with the latter having the highest levels of this inorganic contaminant. 

Brumado also showed a difference between the medians of the two semesters for Pb (*p* = 0.03), wherein in the second semester, the median concentration of this toxic element increased sevenfold. This issue may have been due to the difference between the menus from one semester to the other, the difference in the brands of products used, and the larger supply of food groups that could contribute to a higher percentage of contamination with these elements.

### 3.3. Risk Assessment

All the results found in the analysis of the elements were below the tolerable limits for the methodology used [37,45]. Table 6 shows the study population’s hazard quotients (HQ) in the three proposed scenarios. 

For all the scenarios studied, the HQ did not exceed the limit value of 1 (HQ < 1), which is the ratio between the reference dose (RfD) and the daily intake of the toxic agent evaluated. All the values, including THQ, are well below this parameter; thus, the risk can be classified as negligible [50]. Comparatively, for all the elements, S1 presents the most significant non-cancer risk related to eating school meals, as this group consists of children of a younger age and lower weight. In this case, the food served in schools in Salvador had the highest HQs and, consequently, the highest THQs found, which may be justified by the fact that the average value (in grams) of the meals served was higher in this municipality. Each of these inorganic elements represents a potential risk for the populations under study, as they are a more vulnerable population, given that even small exposures may pose a risk of deleterious effects in the long term. 

One of the study’s limitations was that it was impossible to assess which specific food contributed the highest percentage of the contaminants studied in the meals offered. In addition, due to time demand and budget restrictions, it was not possible to assess the weight of the students in order to obtain more reliable data for calculating the HQs. Another limiting factor of our study was that the leftover food was not evaluated; we assumed that children ingested all the meals provided. 

On the other hand, the fact that the samples were collected in municipalities in different state regions and of varied Brazilian education development indexes (IDEB index, Supplementary Materials Table S1) makes this study more geographically comprehensive and reliable regarding the different realities of the students assisted by the PNAE. In this way, it makes it possible to differentiate between areas with greater or lesser exposure and to promote debate on the importance of contaminant-free food, signaling the need for further studies. It is important to emphasize that the school meals of the Bahian educational system reflect, one way or another, the reality in most regions of Brazil, especially in the northeastern states, and are similar to those in developing countries that have a structured school meal program.

Although school meals do not represent a risk to this group of children, the age and body weight range of children receiving school meals in Brazil vary, and there is a higher risk of exposure for younger children through school meals, combined with food consumed outside of school, and this exposure may exceed the values considered safe by current guidelines.

## 4. Conclusions

This study revealed that, despite public efforts to ensure food safety for children at school, it is crucial to guarantee that meals served at school are not a source of exposure to potentially toxic metals such as As, Cd, and Pb. This study showed that children were exposed to these metals, especially Pb, which, in some school meal samples, were present at higher levels than those found in other studies that evaluated school meals in Brazil and abroad. Despite this exposure, the estimated risk is negligible. Even though the risk estimation is considered negligible, more studies are warranted. There are no safe levels for exposure to those toxic metals, especially lead, for which recent findings show adverse cognitive effects even at blood lead levels below the current recommended value for children.

## Figures and Tables

**Table 1:** Parameters used to estimate the exposure dose to As, Cd, and Pb from the consumption of school meals.

Parameters	S1 (Elementary School)	S2 (Middle School)	S3 (Overall Average)
Duration of exposure (years)	9	3	12
Body weight (Kg)	25.4	51.8	41.2
Exposure frequency (days/year)	202	202	202

**Table 2:** Quality assurance parameters for toxic metal determination in school meals.

	CRM	Certified Value (mg·kg^−1^)	Measured Value (mg·kg^−1^)	LOD (mg·kg^−1^)	LOQ (mg·kg^−1^)	Precision (rSR %)	Accuracy (RE %)
As	Spinach leaves	0.068 ± 0.012	0.067	0.008	0.012	7.73	1.47
Cd	Apple leaves	0.013 ± 0.002	0.012	0.009	0.024	6.80	9.09
Pb	Apple leaves	0.470 ± 0.024	0.471	0.005	0.013	1.61	0.15

**Table 3:** Arsenic content (µg·kg^−1^ *w*/*w*) in school meal samples, by municipality, in two semesters.

Municipalities	Semester	Median	IQR ^1^	Min	Max
Brumado	1st Sem	2	1	<LD	8
	2nd Sem	3	4	2	8
Jacobina	1st Sem	1	<LOD	1	3
	2nd Sem	5	3	3	11
Jaguaripe	1st Sem	2	1	1	6
	2nd Sem	4	3	2	10
Salvador	1st Sem	4	5	<LOD	1
	2nd Sem	4	3	2	10

^1^ IQR: interquartile range.

**Table 4:** Cadmium content (µg·kg^−1^ *w*/*w*) in school meal samples, by municipality, in two semesters.

Municipalities	Semester	Median	IQR ^1^	Min	Max
Brumado	1st Sem	2	2	1	4
	2nd Sem	3	4	2	8
Jacobina	1st Sem	1	<LOD	1	3
	2nd Sem	5	3	3	11
Jaguaripe	1st Sem	2	1	1	6
	2nd Sem	4	3	2	10
Salvador	1st Sem	2	1	1	6
	2nd Sem	4	3	2	10

^1^ IQR: interquartile range.

**Table 5:** Lead content (µg·kg^−1^ *w*/*w*) in school meal samples, by municipality, in two semesters.

Municipalities	Semester	Median	IQR ^1^	Min	Max
Brumado	1st Sem	7	9	1	43
	2nd Sem	42	40	11	96
Jacobina	1st Sem	25	22	1	58
	2nd Sem	38	55	11	101
Jaguaripe	1st Sem	3	8	2	157
	2nd Sem	12	74	4	137
Salvador	1st Sem	3	8	2	39
	2nd Sem	12	74	4	137

^1^ IQR: interquartile range.

**Table 6:** Non-carcinogenic risk quotient associated with school meal consumption for each scenario, S1 (children), S2 (adolescents), and S3 (general population average).

Municipalities	Brumado			Jacobina			Jaguaripe			Salvador		
Scenarios Metals	S1	S2	S3	S1	S2	S3	S1	S2	S3	S1	S2	S3
As–HQ	0.05	0.02	0.05	0.06	0.03	0.04	0.05	0.03	0.03	0.08	0.04	0.05
Cd–HQ	0.02	0.01	0.01	0.02	0.01	0.01	0.02	0.01	0.01	0.01	0.01	0.01
Pb–HQ	0.02	0.01	0.01	0.03	0.01	0.02	0.01	0.0	0.0	0.06	0.03	0.04
THQ	0.09	0.04	0.07	0.11	0.05	0.07	0.08	0.04	0.04	0.15	0.08	0.10

## Data Availability

The original contributions presented in the study are included in the article and supplementary material, further inquiries can be directed to the corresponding author.

## Supplementary Materials

The following supporting information can be downloaded at: https://www.mdpi.com/article/10.3390/foods13101500/s1, Table S1. Characterization of the study municipalities.
