# Supplementary material for: Arsenic, Cadmium, and Lead Levels in School Meals and Their Risk Assessment in Municipalities in Bahia, Brazil
Source: Foods. 2024 May 12;13(10):1500. doi: 10.3390/foods13101500 (PMC11120269; doi:10.3390/foods13101500)
Supplement: Supplementary file 1 [file foods-13-01500-s001.zip › foods-2855916-supplementary.pdf]

Supplementary

Table S1. Characterization of the study municipalities.

Municipalities	IDEB[1] (2017)	IDH[2] (2010)	Geographical location	Population (2019)	Per capita income[3]	No. of enrollments (E. F.)[4]
**Brumado**	6.1	0.656	Southwest Bahia	67.195	R$ 403,00	15.128
**Jacobina**	4.9	0.649	Chapada Diamantina	80.518	R$ 417,00	20.205
**Jaguaripe**	4.3	0.556	Recôncavo Baiano	18.788	R$ 247,00	4.332
**Salvador**	5.3	0.759	East/coast	2.872.347	R$ 973,00	519.719

[1] Basic Education Development Index (INEP, 2017) [2] Human Development Index [3]Average per capita income for the population over 10 years of age who work without taking gender into account in the last census of 2010 (IBGE, 2019); 4Number of students enrolled in elementary school in 2019, according to data from the school census (IBGE, 2019).
